# Effects of oral propranolol on the resistive and pulsatility indices of major abdominal vasculatures in domestic short‐haired cats

**DOI:** 10.1002/vms3.1176

**Published:** 2023-06-13

**Authors:** Shima Amini Masouleh, Mohammad Nasrollahzadeh Masouleh, Bita Vazir, Saied Bokaie

**Affiliations:** ^1^ Department of Clinical Sciences Science and Research Branch Islamic Azad University Tehran Iran; ^2^ Department of Physiology, Science and Research Branch Science and Research Branch Islamic Azad University Tehran Iran; ^3^ Department of Epidemiology Faculty of Veterinary Medicine University of Tehran Tehran Iran

**Keywords:** pulsatility index, resistive index, Doppler ultrasonography, propranolol

## Abstract

**Background:**

No study has been performed regarding the effects of oral administration of propranolol on pulse‐wave spectral Doppler indices of major abdominal vessels in healthy adult cats.

**Objective:**

The objective of this study was to assess the pulse‐wave spectral Doppler indices of abdominal aorta, caudal vena cava, and portal vein in clinically normal adult domestic short‐haired (DSH) cats, before and after propranolol ingestion.

**Methods:**

Twenty intact adult client‐owned DSH cats were evaluated (10 males and 10 females). A duplex Doppler ultrasonography machine with a 10‐MHz frequency linear transducer was used. Peak systolic velocity, end‐diastolic velocity (EDV), resistive index (RI), pulsatility index (PI), and pressure gradient were measured. All the cats received 1 mg/kg of propranolol tablet, and after 2 h, ultrasonography measurements were repeated.

**Results:**

The mean RI of the aorta and caudal vena cava significantly decreased in male cats following oral administration of propranolol after 2 h (*p* = 0.03, *p* = 0.02). In the caudal vena cava, the PI decreased from 2.98 ± 0.62 to 1.15 ± 0.19 post‐propranolol ingestion (*p* = 0.01). The mean EDV in the caudal vena cava of males and portal veins of females significantly decreased after propranolol ingestion (*p* = 0.04, *p* = 0.02).

**Conclusions:**

This study showed that propranolol decreased the PI of the aorta and PI and RI of the caudal vena cava in healthy normal cats 2 h post‐propranolol ingestion at the dosage of 1 mg/kg.

## Graphical abstract

Administration of oral propranolol and monitoring of RI and PI of major abdominal vessels in healthy cats

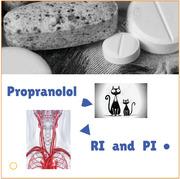

## 1 INTRODUCTION

Pulse‐wave spectral Doppler indices are valuable indicators of vascular resistance and a diagnostic tool in some organs’ diseases in animals and humans (Azizi et al., 2018). The most commonly measured parameters in pulse‐wave spectral Doppler ultrasonography indices are peak systolic velocity (PSV), end‐diastolic velocity (EDV), resistive index (RI), and pulsatility index (PI) (Penninck & d'Anjou, 2015).

Measurements of RI and PI of major hepatic and splenic vessels have been used as a biomarker in humans with hepatic diseases (Baikpour et al., 2020; Tana et al., 2016). Hepatic artery's RI and portal venous PI have been evaluated in patients with non‐alcoholic fatty liver disease and normal subjects, and a significant reduction in both RI and PI was noted in patients with high‐risk non‐alcoholic fatty liver disease (Baikpour et al., 2020; Tana et al., 2016). In patients with hepatic cirrhosis, RI significantly increased compared to normal participants (Sacerdoti et al., 1995). Portal vein thrombosis in cirrhotic patients has been noted to increase both RI and PI compared to the control group (Sacerdoti et al., 1995). Moreover, the RI and PI of the renal artery in patients with hepatic cirrhosis have been reported to have positive correlations with the severity of hepatic disease (Popov et al., 2012).

Pulse‐wave spectral Doppler indices such as RI and PI play a role in the diagnosis of some diseased states in dogs and cats with kidney diseases (Hanamura et al., 2012; Rivers et al., 1997). In cats with renal disease, a positive linear correlation has been noted between RI, PI, and serum creatinine level (Novellas et al., 2010). Persistent hypertension is another risk factor for renal diseases in cats. It can damage the kidney and result in glomerular diseases, but no correlation has been found between either renal PI or RI and systemic blood pressure in cats (Novellas et al., 2010). A rise in PI and RI has been reported in dogs and cats with renal diseases (Constantinescu et al., 2015; Novellas et al., 2010).

Propranolol is a non‐selective beta‐adrenergic receptor blocker agent (acting on both beta‐1 and beta‐2 adrenergic receptors). It is used for the treatment of hypertension and cardiac arrythmia (Propranolol, 2012). Propranolol decreases heart rate, cardiac conduction, and blood pressure (Papich, 2020). In cats, propranolol should be used cautiously at a lower dosage in hyperthyroid states, as it can decrease clearance or increase absorption in hyperthyroid cats. The routine dosage of propranolol in cats is 0.4–1.2 mg/kg every 8 h (Papich, 2020).

This study aimed to assess the pulse‐wave spectral Doppler indices of the abdominal aorta, caudal vena cava, and portal vein in clinically normal adult domestic short‐haired (DSH) cats, before and after propranolol ingestion. To the best of the authors’ knowledge, this is the first report regarding the effects of oral administration of propranolol on pulse‐wave spectral Doppler indices of major abdominal vessels in healthy adult DSH cats.

## 2 MATERIALS AND METHODS

Twenty intact adult client‐owned DSH cats were evaluated in this study (10 males and 10 females). All the animals underwent a full physical examination. Results of complete blood count, biochemistry profile, urinalysis, and abdominal ultrasonography were evaluated before the beginning of the study. No animal had a history of any cardiac or systemic disease, and they were all negative for Feline Leukemia Virus (FeLV) and Feline immunodeficiency virus (FIV) based on the medical records.

A duplex Doppler ultrasonography machine (Mindray M5 Veterinary Ultrasound Machine, Mindray Ltd., China) with a 10‐MHz frequency linear transducer was used in this study. Cats were on dorsal recumbency at the time of ultrasonographic examination. The hair of the abdominal area of all the cats was clipped, and the ultrasonography gel (Smart care Ultrasound Gel, Mumbai, India) was applied. After visualization of each major vasculature (abdominal aorta, caudal vena cava, portal vein, and hepatic vein) in B‐mode ultrasonography, colour Doppler mapping was used. During ultrasonography, some adjustments were applied to transducers and the machine based on the animal's body condition to have high‐quality images.

A small value of wall filter and pulse repetition frequency was used that displaced the flow with aliasing. PSV and EDV were measured manually (using calipers) from the recorded spectra. PI, RI, mean blood velocity, and pressure gradient (PG) were determined automatically by the ultrasound machine. All the cats received 1 mg/kg of propranolol tablet (Propranolol tablet, Hakim Pharmaceutical Co., Tehran, Iran), and after 2 h, ultrasonography measurements were repeated.

The same investigator (Shima Amini Masouleh) performed all the measurements at the same time of the day (8–11 AM). Cardiac auscultation was performed by an internist, and blood pressure was measured after propranolol administration up to 2 h.

Statistical analysis was performed using the SPSS statistical software program (SPSS Statistic ver. 26, IBM, Chicago, IL, USA). The one‐sample Kolmogorov–Smirnov test was used to check normality. The measured parameters were compared before and after drug ingestion via a one‐way analysis of variance. Independent samples *t*‐test was used to compare the obtained values between males and females. *p*‐Values <0.05 were considered statistically significant. The data are presented here as mean ± SD.

## 3 RESULTS

Mean ± SD body weight and age of cats were 3.5 ± 1.5 kg and 3.8 ± 1.6 years, respectively. Mean ± SD RI of the aorta, caudal vena cava, and portal vein before propranolol administration were 0.85 ± 0.05, 0.96 ± 0.04, 0.89 ± 0.07 in males and 0.81 ± 0.03, 0.95 ± 0.05, 0.78 ± 0.07 in females, respectively. Two hours after ingestion of propranolol, the mean ± SD RI of the aorta, caudal vena cava, and portal vein before propranolol administration was changed to 0.63 ± 0.09, 0.71 ± 0.1, 1.07 ± 0.24 in males and 0.77 ± 0.04, 0.95 ± 0.05, 0.71 ± 0.08 in females, respectively. The mean RI of the aorta and caudal vena cava significantly decreased in male cats following oral administration of propranolol after 2 h (*p* = 0.03, *p* = 0.02).

Mean ± SD PI of the aorta in males increased from 1.56 ± 0.10 to 1.86 ± 0.44 after propranolol ingestion (*p* = 0.5). In the portal vein, a rise in PI was observed in males after 2 h from 1.83 ± 0.30 to 2.42 ± 0.57 (*p* = 0.4). In the caudal vena cava, the PI decreased from 2.98 ± 0.62 to 1.15 ± 0.19 post‐propranolol ingestion (*p* = 0.01).

In females, the mean ± SD PI of the aorta, caudal vena cava, and portal vein before and after propranolol administration were 1.60 ± 0.09, 3.65 ± 0.52, and 1.27 ± 0.17 and 1.62 ± 0.11, 3.54 ± 0.46, and 1.12 ± 0.14, respectively (*p* = 0.7, *p* = 0.2, *p* = 0.1).

There was no statistical difference before and after drug ingestion in any cat on any occasion in the mean of PSV values. The mean PG significantly increased in the portal vein of male cats from 0.27 ± 0.13 cm/s to 0.59 ± 0.12 cm/s (*p* = 0.007) after propranolol ingestion. Mean EDV in the caudal vena cava of males and portal veins of females significantly decreased after propranolol ingestion (*p* = 0.04, *p* = 0.02).

Results of PSV, EDV, RI, PI, and PG are presented in Tables 1 and 2.

**TABLE 1:** Mean ± SD of peak systolic velocity (PSV), end‐diastolic velocity (EDV), resistive index (RI), pulsatility index (PI), and pressure gradient (PG) in 20 domestic short‐haired cats before propranolol ingestion.

	Aorta		Caudal vena cava		Portal vein	
Parameters	Male	Females	Male	Females	Male	Females
PSV	2.38 ± 0.15	2.49 ± 0.13	−2.43 ± 0.57	−2.39 ± 0.46	−1.45 ± 0.51	−1.07 ± 0.16
EDV	0.62 ± 0.09	0.61 ± 0.07	−0.03 ± 0.09	−0.07 ± 0.09	−0.09 ± 0.13	0.42 ± 0.09
RI	1.56 ± 0.10	1.60 ± 0.09	2.98 ± 0.62	3.65 ± 0.52	1.83 ± 0.30	1.27 ± 0.17
PI	0.85 ± 0.05	0.81 ± 0.03	0.96 ± 0.04	0.95 ± 0.05	0.89 ± 0.07	0.78 ± 0.07
PG	0.41 ± 0.05	0.52 ± 0.06	0.22 ± 0.02	0.21 ± 0.01	0.27 ± 0.13	0.35 ± 0.10

**TABLE 2:** Mean ± SD of Peak systolic velocity (PSV), end‐diastolic velocity (EDV), resistive index (RI), pulsatility index (PI), and pressure gradient (PG) in 20 domestic short‐haired cats after 2 hours after 1 mg/kg of propranolol ingestion.

	Aorta		Caudal Vena Cava		Portal Vein	
Parameters	Male	Females	Male	Females	Male	Females
PSV	2.82 ± 0.30	2.39 ± 0.13	−2.07 ± 0.26	−2.35 ± 0.42	−1.20 ± 0.77	−1.19 ± 0.14
EDV	1.22 ± 0.37	0.60 ± 0.05	−0.69 ± 0.21	−0.04 ± 0.05	−0.29 ± 0.35	0.20 ± 0.07
RI	1.86 ± 0.44	1.62 ± 0.11	1.15 ± 0.19	3.54 ± 0.46	2.42 ± 0.57	1.12 ± 0.14
PI	0.63 ± 0.09	0.77 ± 0.04	0.71 ± 0.1	0.95 ± 0.05	1.07 ± 0.24	0.71 ± 0.08
PG	1.40 ± 0.57	0.51 ± 0.06	0.26 ± 0.06	0.22 ± 0.02	0.59 ± 0.12	0.29 ± 0.09

## 4 DISCUSSION

This study demonstrated significant alterations in RI, PI, EDV, and PG in the aorta, caudal vena cava, and portal vein post‐propranolol administration in healthy adult cats. Propranolol ingestion significantly decreased RI after 2 h compared to baseline values in the aorta. This significant finding after oral medication of propranolol on the Doppler ultrasonography of the aorta was observed in only male cats. RI, PI, and EDV significantly dropped in the caudal vena cava of the studied cats post‐propranolol ingestion. EDV declined in both males and females; however, RI and PI were only reduced in males. The increase in PG was the only significant finding in the portal vein in the studied cats after 2 h of propranolol ingestion.

Propranolol is well absorbed after oral administration, yet the rapid‐first pass effect across the liver decreases systemic bioavailability. Propranolol is metabolized by the liver, and its active metabolite in humans is 4‐hydroxypropranolol (Plumbs). The mean half‐life of propranolol following intravenous administration has been reported as 35 min in cats and 1 h in dogs (Kates et al., 1979; Weidler et al., 1979).

Effects of propranolol on vascular pulse‐wave spectral Doppler indices have been evaluated in humans and various animals. In humans, propranolol has been reported to reduce the RI of renal artery (Sacerdoti et al., 1995). Reportedly, it increases the aortic pulse‐wave velocity of Wistar rats (Lampropoulos et al., 2012). Meanwhile, systolic and diastolic blood pressure has been reported to significantly drop after oral administration of propranolol for 3 months (Lampropoulos et al., 2012). The hepatic arterial vascular resistive index of rats has been reported to decrease after propranolol administration (Calès et al., 1985). In rabbits, the blood flow velocity of the common carotid artery has been reported to decline up to 14 min after propranolol injection (Jung et al., 2000). The results of the present study agree with previous findings on rats, rabbits, and humans.

Pulse‐wave spectral Doppler indices have been reported to have diagnostic and prognostic values in animals and humans. PI is more sensitive compared to RI in the case of diagnosing abnormal waveforms in ocular and renal vasculatures. The reason is that PI shows a mean velocity during a single cycle (Novellas et al., 2007).

PI and RI have been evaluated in the case of renal diseases in dogs and cats. Elevations in RI and PI in both species diagnosed with renal insufficiency have been reported (Novellas et al., 2010). Severe acute anaemia has been implicated as an influencing factor on RI and PI of renal arteries in dogs (Koma et al., 2006).

In humans, fatty liver (non‐alcoholic or alcoholic form) is a common disease. Colour Doppler ultrasonography of liver vasculature and venous PI is a vital non‐invasive biomarker in high‐risk fatty liver disease (Baikpour et al., 2020). PI is lower in patients with fatty liver (0.19) compared to normal subjects (0.32) in high‐risk patients (Baikpour et al., 2020).

There were some limitations to this study. Since all the cats were healthy, it was not possible to use propranolol as a long‐term medication. It would be beneficial to measure pulse‐wave spectral Doppler indices of major abdominal vasculatures under long‐term administration of oral propranolol. Blood pressure and heart rate were not recorded as data in this study, which is the second limitation of this study.

In conclusion, this study showed that propranolol decreased the PI of the aorta and PI and RI of caudal vena cava in healthy normal cats 2 h post‐propranolol ingestion at the dosage of 1 mg/kg.

## AUTHOR CONTRIBUTIONS

*Data curation, investigation, project administration, and writing—original draft*: Shima Amini Masouleh. *Investigation, methodology, supervision, and writing—review and editing*: Mohammad Nasrollahzadeh Masouleh. *Conceptualization, methodology, project administration, resources, and visualization*: Bita Vazir. *Data curation, formal analysis, methodology, software, and validation*: Saied Bokaie.

## CONFLICT OF INTEREST STATEMENT

The authors declare no conflict of interest.

## FUNDING INFORMATION

The authors received no financial support for the research, authorship and/or publication of this article.

### ETHICS STATEMENT

This study was conducted based on Iranian ethical codes for studies on animals and approved by the Ethical Review Committee of the Islamic Azad University (IAU) Science and Research Branch.

### PEER REVIEW

The peer review history for this article is available at https://publons.com/publon/10.1002/vms3.1176.

## Data Availability

Data are available upon request from the corresponding author.
